# The Nexus of Sleep Disorders and Violence in Patients with Schizophrenia: What do the Data Say?

**DOI:** 10.1192/j.eurpsy.2024.1606

**Published:** 2024-08-27

**Authors:** K. Razki, A. Larnaout, C. Najar, S. Ben Aissa, R. Lansari

**Affiliations:** Psychiatry department, razi hospital, manouba, Tunisia

## Abstract

**Introduction:**

One of the common symptoms of schizophrenia is sleep disturbances, which can have a significant impact on the quality of life of patients. Several studies suggest the existence of a complex link between sleep disorders and agressive behavior in patients with schizophrenia.

**Objectives:**

to determine the impact of sleep disorders on aggressive behavior in patients with schizophrenia.

**Methods:**

We conducted a cross-sectional, descriptive, and analytical study that took place over a period of one month (from 1st to 31nd March 2023) with patients consulting the post-cure of Psychiatry Service D at Razi Hospital, Tunisia. We included patients diagnosed with schizophrenia according to DSM5, and stabilized on a psychiatric plan. We used the Pittsburgh Sleep Quality Index (PSQI) to assess sleep quality over a period of one month. The Buss & Perry Aggression Questionnaire (QABP) was used to measure aspects of aggression. We used the Adult Social Relationships Scales (ASRS), part of the National Institute of Health (NIH) toolkit, assessing six domains of social relationships: perceived rejection, perceived hostility, loneliness, friendship, instrumental support and emotional support.

**Results:**

We collected data from 40 male patients with a mean age of 42.5 ± 14.02. The mean global PSQI score was 9.23 ± 4.58.

Ten patients were on typical antipsychotics, 25 patients were on atypical antipsychotics, and the remaining five patients were on a combination therapy (both atypical and typical antipsychotics). Regarding the use of benzodiazepines, 34 patients were taking lorazepam at a dose of 2.5 to 5 mg per day. he mean QABP global score was 45 ± 12.3 out of 72.

For the subjective evaluation, all patients self-reported feeling “irritable,” “dysphoric,” “unable to communicate with others,” and “wanting to break objects” when they experienced insomnia.

We found a statistically significant association between QABP and daytime dysfunction (p=0.003).

The overall PSQI score was higher, and statistically significantly associated, in patients who reported low emotional support (p=0.018) and perceived social rejection (p=0.04).

**Conclusions:**

An integrated approach that includes the evaluation of sleep disorders, as well as the prevention and management of violence, can play a key role in the overall improvement of the mental health of patients with schizophrenia.

**Disclosure of Interest:**

None Declared

